# IL-21 regulates SOCS1 expression in autoreactive CD8^+^ T cells but is not required for acquisition of CTL activity in the islets of non-obese diabetic mice

**DOI:** 10.1038/s41598-019-51636-5

**Published:** 2019-10-25

**Authors:** Andrew P. R. Sutherland, Kate L. Graham, Michelle Papadimitriou, Gaurang Jhala, Prerak Trivedi, Tara Catterall, Stacey Fynch, Thomas W. H. Kay, Helen E. Thomas

**Affiliations:** 1 St Vincent’s Institute of Medical Research, Melbourne, Australia; 2 The University of Melbourne, Department of Medicine, St. Vincent’s Hospital, Fitzroy, Victoria Australia

**Keywords:** Autoimmunity, Interferons, Interleukins, Cytotoxic T cells, Type 1 diabetes

## Abstract

In type 1 diabetes, maturation of activated autoreactive CD8^+^ T cells to fully armed effector cytotoxic T lymphocytes (CTL) occurs within the islet. At present the signals required for the maturation process are poorly defined. Cytokines could potentially provide the necessary “third signal” required to generate fully mature CTL capable of killing insulin-producing β-cells. To determine whether autoreactive CTL within islets respond to cytokines we generated non-obese diabetic (NOD) mice with a reporter for cytokine signalling. These mice express a reporter gene, hCD4, under the control of the endogenous regulatory elements for suppressor of cytokine signalling (SOCS)1, which is itself regulated by pro-inflammatory cytokines. In NOD mice, the hCD4 reporter was expressed in infiltrated islets and the expression level was positively correlated with the frequency of infiltrating CD45^+^ cells. SOCS1 reporter expression was induced in transferred β-cell-specific CD8^+^ 8.3T cells upon migration from pancreatic draining lymph nodes into islets. To determine which cytokines induced SOCS1 promoter activity in islets, we examined hCD4 reporter expression and CTL maturation in the absence of the cytokine receptors IFNAR1 or IL-21R. We show that IFNAR1 deficiency does not confer protection from diabetes in 8.3 TCR transgenic mice, nor is IFNAR1 signalling required for SOCS1 reporter upregulation or CTL maturation in islets. In contrast, IL-21R-deficient 8.3 mice have reduced diabetes incidence and reduced SOCS1 reporter activity in islet CTLs. However IL-21R deficiency did not affect islet CD8^+^ T cell proliferation or expression of granzyme B or IFNγ. Together these data indicate that autoreactive CD8^+^ T cells respond to IL-21 and not type I IFNs in the islets of NOD mice, but neither IFNAR1 nor IL-21R are required for islet intrinsic CTL maturation.

## Introduction

Type 1 diabetes (T1D) is an organ specific autoimmune disease that results from the destruction of insulin producing β-cells in the pancreatic islets. CD8^+^ T cells are the main mediators of β-cell destruction in both non-obese diabetic (NOD) mice and humans^1,2^. In T1D, autoreactive CD8^+^ T cells become activated in the pancreatic draining lymph node (PLN) and then migrate to the islets via the circulation where they accumulate. Maturation of activated autoreactive CD8^+^ T cells to fully armed effector cytotoxic T lymphocytes (CTL) occurs within the islets^3^ and involves increased expression of cytotoxic machinery including granzyme B, IFNγ and CD107a and enhanced CTL function. While antigen presentation by β-cells is required to promote CD8^+^ T cell proliferation and accumulation within islets^4,5^, maturation of CTL effector function occurs independently of antigen presentation by β-cells^3^. These islet intrinsic proliferation and differentiation events have been termed the “mezzanine response”^5^. In this context, cytokines produced within the islets provide proliferative signals and it is possible that they may also provide a “third signal” required to generate fully mature and functional CTLs capable of killing β-cells^6,7^.

Cytokines are essential regulators of CD8^+^ T cell activation, differentiation and survival^8–10^. Previously published studies suggest that type I IFNs and/or IL-21 could potentially provide the islet intrinsic maturation signal. Type I IFNs are implicated in the pathogenesis of T1D^11–14^ and have been shown to influence mouse and human CTL maturation^11,15^. IL-21 and IL-21R are essential for the development of autoimmune diabetes in NOD mice^16–19^. Strikingly, transgenic expression of type I IFNs or IL-21 in pancreatic β-cells leads to the development of autoimmune diabetes^18,20^ suggesting that both are likely to play important roles in islets. IL-21 is an important growth factor for T cells in situations where there is chronic antigen exposure, such as chronic viral infection or autoimmunity^21,22^. IL-21 induces expression of transcription factors important for CD8^+^ CTL maturation including T-bet and BATF3^23,24^.

Type I IFNs and IL-21 both signal through the JAK-STAT pathway that is activated in response to numerous cytokines and has essential roles in the differentiation and survival of effector and memory CD8^+^ T cells^25,26^. STAT1 is activated in CD8^+^ T cells after stimulation by cytokines including type I and II interferons (IFNs) and regulates transcriptional responses that control clonal expansion and effector differentiation^27,28^. Further STAT1 activation induces the expression of suppressor of cytokine signalling (SOCS) proteins such as SOCS1, which are negative regulators of JAK-STAT signalling^29,30^. These pathways are important regulators of autoimmune diabetes development in mouse models. STAT1 deficient NOD mice are completely protected from the development of diabetes and treating NOD mice with JAK inhibitors prevents autoimmune diabetes^31,32^.

At present, mechanisms of islet intrinsic CTL maturation and the identity of any potential cytokine “third signal” remain elusive. To address this question we generated novel NOD SOCS1 reporter mice that report pro-inflammatory cytokine signalling^33^ and used these to determine the influence of SOCS1 activating cytokines in islet CD8^+^ CTL differentiation processes.

## Materials and Methods

### Mice

NOD/Lt, NOD8.3, NOD/IFNAR1^34^ and NOD/IL-21R^35^ have previously been described. Generation of SOCS1/hCD4 reporter mice carrying a modified hCD4 gene immediately 3′ of a SOCS1^lox^ gene in the endogenous SOCS1 locus was previously described^33^. These mice were backcrossed for 10 generations to the NOD genetic background and maintained as heterozygous NOD.SOCS1^lox/+^Cre^+^. Genomic DNA from the 10th generation was processed for Illumina mouse medium density linkage panel containing 1,449 single nucleotide polymorphism (SNP) loci by The Centre for Applied Genomics (Toronto, ON, Canada). The Jackson Laboratory Mouse Genome Informatics (MGI) and National Center for Biotechnology Information (NCBI) databases were used to identify strain differences. The congenic interval around the *Socs1* gene in the backcrossed mice was between and including Chr16:5,029,200 (rs4152838; GRCm38/mm10 assembly) and Chr16:51,637,127 (rs4187143). All mice were bred and housed in microisolator cages under specific pathogen-free conditions at the St Vincent’s Hospital BioResources Centre. All animal care and experiments were approved by the St Vincent’s Animal Ethics Committee. All animal studies were performed following the guidelines of the institutional animal ethics committee and the experiments were carried out in accordance with the approved guidelines.

### Immunohistochemistry

5 μm frozen sections were prepared from 3 levels (200 μm apart), acetone fixed and stained with guinea pig anti-insulin followed by horseradish peroxidase-conjugated anti-guinea pig Ig (Dako Cytomation, Carpenteria, CA)^36^. Serial sections were stained with biotinylated anti-hCD4 and anti-mCD8 (both BD Biosciences) followed by incubation with Vectastain Elite ABC reagent. Stains were developed with Sigma Fast 3,3′-Diaminobenzidine peroxidase substrate followed by counterstaining with haemotoxylin. Images were photographed with a Leica microscope fitted with a Leica camera at a magnification of 200x.

### Antibodies

Antibodies used for flow cytometric analysis were anti-mouse as follows: CD8 (53-6.7, Biolegend), IFNγ (XMG1.2, Ebioscience), granzyme B (16G6, Ebioscience), except anti-human CD4 (RPA-T4, Biolegend).

### Analysis of diabetes

Mice were monitored for diabetes by measurement of urinary glucose levels with Diastix (Bayer Diagnostics). Mice suspected of hyperglycaemia were further tested on two consecutive days and those with three positive tests were considered diabetic. Blood glucose levels (≥15 mmol/L) were confirmed using Advantage II Glucose Strips (Roche).

### CFSE labelling, cell transfer and islet isolation

CD8^+^ T cells from NOD8.3 mice were labelled with carboxy-fluorescein succinimidyl ester (CFSE) as previously described^37^. Cells were resuspended at 2.5 × 10^7^/ml in PBS, and 200 μl was injected i.v. into the tail vein of recipient mice. After 5 days the mice were sacrificed, and the inguinal lymph node (ILN), pancreatic lymph node (PLN) and islets were harvested. Mouse islets were isolated as described previously^38^.

### Restimulation culture and flow cytometry

Lymph nodes harvested from recipient mice were prepared as single-cell suspensions. Islets were dispersed to single cells with 0.1 mg/ml bovine trypsin (Calbiochem) and 2 mM EDTA for 5 minutes at 37 °C and gentle pipetting. Dispersed islets were washed in RPMI 1640 medium containing penicillin/streptomycin, 2 mM glutamine, nonessential amino acids 50 µM mercaptoethanol and 10% fetal calf serum (complete RPMI; Gibco) and allowed to recover for 1–2 hours at 37 °C in 5% CO_2_. For IFNγ expression analyses cells were cultured *in vitro* with IGRP_206-214_ peptide (VYLKTNVFL, Auspep) for 6 hours. For cell surface staining cells were harvested and resuspended in PBS containing 0.5% BSA and stained using standard procedures. Intracellular staining was performed using the Cytofix/Cytoperm Plus kit (BD Biosciences, San Jose, CA). Data was collected using a BD Fortessa flow cytometer (BD Biosciences) and subsequently analysed on FlowJo software (version 8.7.3).

### ^51^Cr release assay

CFSE labelled CD8^+^ 8.3T cells were isolated from mouse pancreatic lymph nodes and islets 5 days after adoptive transfer and CD45^+^ CD8^+^ CFSE diluted cells were sorted using a FACS Aria (BD Biosciences). ^51^Cr release assays were performed as previously described^39^. P815 mastocytoma cells were loaded with 200 µCi [^51^Cr] sodium chromate (Amersham Pharmacia Biotech) and IGRP_206-214_ peptide. Target P815 cells were incubated with sorted CD8^+^ T cells at 5:1 effector:target ratio in triplicate for 16 hours at 37 °C. Medium alone or 2% Triton X-100 was added to a set of target cells to determine spontaneous and total cell lysis respectively. The radioactivity of harvested supernatant was measured on a gamma counter (Perkin-Elmer). Specific ^51^Cr release was calculated as percent lysis = (test cpm − spontaneous cpm)/(total cpm-spontaneous cpm) × 100.

### Statistical analysis

Data are presented as the mean ± SEM. Statistical significance was determined using one-way ANOVA with Sidak’s post-test for multiple comparisons and Student’s T-test. Diabetes incidence was analysed using Log-rank (Mantel-Cox) test. Statistical significance values indicated as follows: p < 0.05 (*), p < 0.01 (**) and p < 0.001 (***). Analysis was performed using GraphPad Prism 5.0 Software.

## Results

### Development of a reporter for cytokine signalling in islets

We previously generated mice on the C57BL/6 background with a reporter for SOCS1 promoter activity^33^. These mice have the *Socs1* gene replaced with a modified *Socs1* gene flanked by LoxP sites (SOCS1^lox^) and a 3′ reporter gene. The reporter is human CD4 (hCD4) containing a F43I mutation and intracellular truncation abrogating its function^40^. When SOCS1^lox^ is deleted with Cre, cell surface hCD4 is expressed in place of SOCS1 and is a reporter of SOCS1 promoter activity. SOCS1^lox^ mice were bred with CMV-Cre mice with Cre expression in early embryogenesis and thus SOCS1 deletion occurred in all cells in the body^41^. SOCS1^lox/+^.Cre^+^ mice were then backcrossed to the NOD genetic background and maintained as SOCS1^lox/+^ heterozygotes (called NOD.SOCS1/hCD4), which resulted in healthy mice free of any disease normally associated with homozygous SOCS1 deletion^42^.

### SOCS1 reporter activity in pancreatic islets of NOD mice

**Figure 1:**
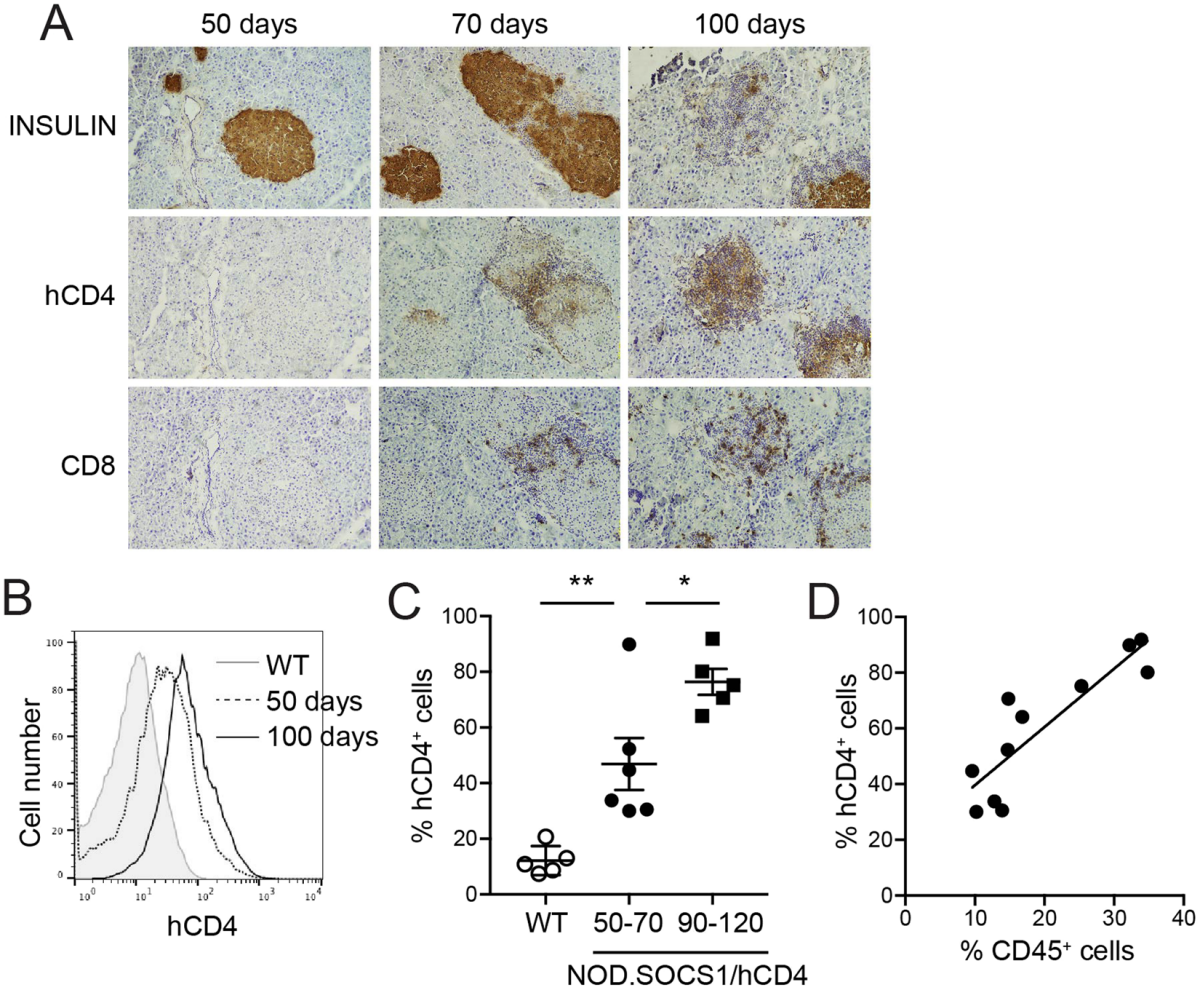
SOCS1 reporter expression in islets of NOD mice. (**A**) Serial pancreas sections from 50-, 70 and 100-day old female NOD.SOCS1/hCD4 mice were stained with antibodies to insulin, human CD4 (hCD4) and murine CD8. Representative sections from n = 3 mice/age are shown. Magnification 200x. (**B**–**D**) Islets were isolated from female NOD.SOCS1/hCD4 mice or wild-type littermates at 50–70 days of age and 90–120 days of age. The proportion of CD45^+^ cells and hCD4^+^ cells in the islets was determined by flow cytometry. (**B**) Representative histograms of hCD4 staining in CD45^−^ islet cells of wild-type (WT) and NOD.SOCS1/hCD4 mice at 50- and 100-days of age. (**C**) Pooled data showing mean ± SEM of the %CD45^−^hCD4^+^ cells in the islets of individual mice (n = 5–6/group). **p = 0.0056, *p = 0.016 one-way ANOVA with Sidak’s post-test for multiple comparisons. (**D**) Correlation of %CD45^−^hCD4^+^ cells with %CD45^+^ cells in the islets of NOD.SOCS1/hCD4 mice. R^2^ = 0.763, p = 0.0004, linear regression analysis.

SOCS1 reporter activity was examined in the islets of NOD.SOCS1/hCD4 mice during progression to diabetes by immunohistochemistry and flow cytometry. There was no hCD4 expression in islets of young mice without insulitis (Fig. 1A). In mice with insulitis, hCD4 expression was observed in insulin^+^ β-cells in close proximity to immune cells, and in the immune cells themselves (Fig. 1A). More cells were hCD4^+^ in islets with more insulitis. We used flow cytometry to quantify hCD4 expression in the islets of NOD.SOCS1/hCD4 mice. The proportion of β-cells expressing hCD4 increased with age, and correlated positively with the proportion of CD45^+^ immune cells in the islets (Fig. 1B–D). These data indicate that cytokines that induce SOCS1 expression are present in the islets of NOD mice, and SOCS1 reporter activity increases as insulitis increases.

### CTL effector function is acquired in the islet

**Figure 2:**
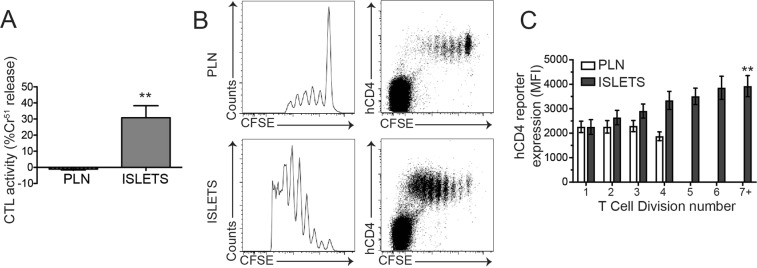
SOCS1 reporter expression is increased during CD8^+^ CTL differentiation in NOD islets. (**A**) Pancreatic lymph nodes and islets were isolated 5 days after 8.3 splenocyte transfer, CFSE positive cells were isolated by cell sorting and CTL activity quantified *in vitro*. Data show mean + SEM from 3 independent experiments. **p < 0.01. (**B**) Representative plots of pancreatic lymph nodes (PLN) and islets isolated 5 days after 8.3/hCD4 splenocyte transfer showing expression of hCD4 and CFSE dilution. (**C**) Expression of hCD4 (mean fluorescence intensity, MFI) on CD8^+^CFSE^+^ T cells for each cell division was calculated using CFSE dilution profiles. Data show mean ± SEM for n = 6 mice from 3 independent experiments. **p < 0.01, one-way ANOVA comparing division 1 and division 7.

We have previously demonstrated the existence of islet intrinsic CD8^+^ CTL differentiation mechanisms in NOD mice^3^. In these studies, CTL maturation was quantified by flow cytometry of cytotoxic markers including IFNγ, granzyme B and CD107a expression. To confirm that this corresponded to a *bone fide* increase in CTL activity we performed *in vitro* CTL assays. NOD8.3T cells were labelled with CFSE and transferred to 12–15 week old NOD recipients. After 5 days CD8^+^ CFSE^+^ T cells were isolated from pancreatic draining lymph nodes and islets and used in a CTL assay, against P815 cells loaded with IGRP_206-214_ peptide. We observed a potent increase in CTL activity in 8.3 cells isolated from islets compared to pLN (Fig. 2A), demonstrating that functional maturation of CD8^+^ CTLs occurs in islets in accordance with our previous experiments^3^.

### CTLs within islets respond to SOCS1 activating cytokines

We next sought to determine whether cytokine stimulation may be an important part of this CD8^+^ T cell maturation in islets. We crossed NOD8.3 TCR transgenic mice to NOD.SOCS1/hCD4 reporter mice to generate NOD8.3/hCD4 mice that express hCD4 on the cell surface after stimulation with SOCS1 activating cytokines. CFSE labelled 8.3 T cells expressing the SOCS1/hCD4 reporter were then adoptively transferred into NOD mice. After 5 days, SOCS1/hCD4 reporter expression (mean fluorescence intensity, MFI) was quantified for each cell division number based on CFSE dilution as an indication of SOCS1 promoter activity (Fig. 2B, Supplementary Fig. 1). Our data show that hCD4 expression was increased in transferred cells compared to endogenous cells, and did not change with cell division in pancreatic lymph nodes. In contrast, in the islets hCD4 expression gradually increased as the transferred cells divided (Fig. 2C), with an approx. 2-fold increase in expression level between division 1 and division 7 (p < 0.01). These data suggest that autoreactive CD8^+^ 8.3T cells upregulate the SOCS1 reporter in the islets of NOD mice, indicating that they respond to proinflammatory cytokines in the islet milieu.

Both type I IFNs and IL-21 regulate SOCS1 expression in T cells^43,44^ and both regulate the development of type 1 diabetes in NOD mice. Thus we next tested whether type I IFNs or IL-21 were mediators of the observed SOCS1 induction in islet CD8^+^ T cells and/or were required for CTL differentiation and diabetes development.

### CD8^+^ CTL differentiation within islets does not require IFNAR1

**Figure 3:**
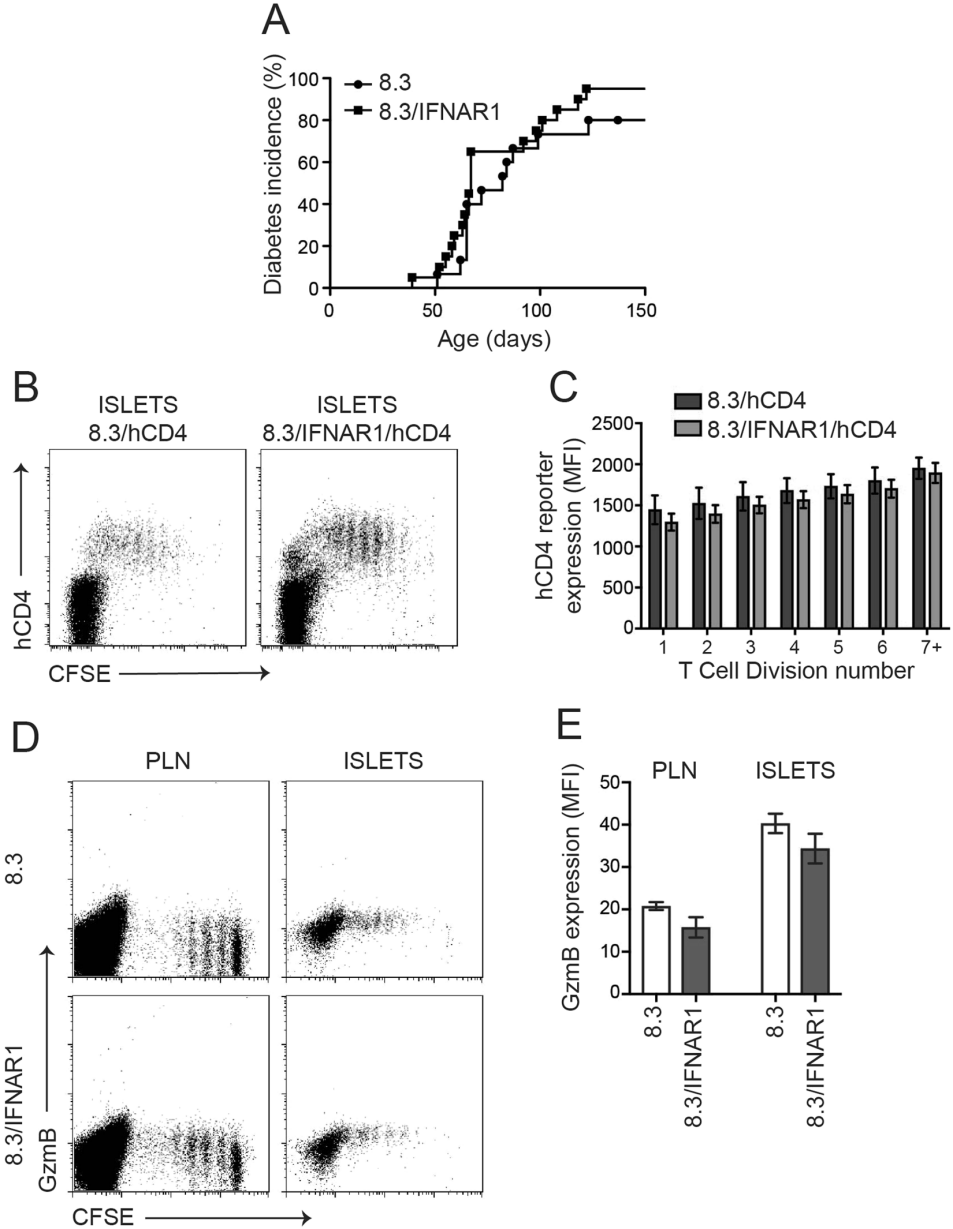
IFNAR1 is not required for SOCS1 upregulation or CTL differentiation in 8.3 CD8^+^ T cells in NOD islets. (**A**) Diabetes incidence in 8.3/IFNAR1 and 8.3 controls (n = 15–20 mice per group). (**B**) Representative plots of islets isolated 5 days after 8.3/hCD4 or 8.3/IFNAR1/hCD4 splenocyte transfer showing expression of hCD4 and CFSE dilution. (**C**) Expression of hCD4 (MFI) on CD8^+^CFSE^+^ T cells for each cell division calculated using CFSE dilution profiles. Data show mean ± SEM for n = 7–8 mice from 3 independent experiments. Difference between MFI in division 1 and MFI in division 7 for wild-type (p = 0.03) and IFNAR1 (p = 0.0003) cells, one-way ANOVA with Sidak’s post-test for multiple comparisons. (**D**) Representative plots of pancreatic lymph nodes (PLN) and islets isolated 5 days after 8.3 or 8.3/IFNAR1 splenocyte transfer showing expression of granzyme B and CFSE dilution. (**E**) Mean granzyme B expression (MFI) on CD8^+^ T cells in pancreatic lymph nodes and islets. Data show mean ± SEM for n = 4–5 mice from 3 independent experiments. Difference between 8.3 and 8.3/IFNAR1 not statistically significant.

Type I IFNs were recently shown to induce CTL effector function *in vitro* in human CTL avatars recognizing the islet antigen IGRP^15^. Also, IFNAR1-deficient CD8^+^ T cells displayed impaired expansion and effector CTL differentiation in viral models^45^. Further type I IFNs activate SOCS1 expression in T cells^46^. Thus we tested whether type I IFNs are required for CD8^+^ CTL differentiation in pancreatic islets. We crossed NOD8.3 mice to NOD/IFNAR1 knockout mice to generate NOD8.3 mice deficient for IFNAR1 (NOD8.3/IFNAR1). The incidence of autoimmune diabetes in female NOD8.3/IFNAR1 mice was indistinguishable from wildtype NOD8.3 mice (Fig. 3A).

We then crossed NOD8.3/hCD4 mice to NOD/IFNAR1 mice to generate SOCS1/hCD4 reporter mice deficient for IFNAR1 (NOD8.3/IFNAR1/hCD4). CFSE labelled T cells from NOD8.3/hCD4 or NOD8.3/IFNAR1/hCD4 mice were adoptively transferred into 12–15 week old NOD mice and analysed on day 5 post-transfer (Fig. 3B). As per our previously published analyses, there were no observable proliferative defects in IFNAR1 deficient 8.3 cells after transfer (data not shown)^34^. SOCS1/hCD4 reporter expression in the transferred 8.3 T cells was not affected by the absence of IFNAR1 in PLN or pancreatic islets (Fig. 3C). Similarly granzyme B upregulation in islet CD8^+^ T cells was not affected by the absence of IFNAR1 (Fig. 3D,E), indicating that it is unlikely that type I IFNs play a non-redundant role in islet intrinsic CTL differentiation in NOD mice.

### CD8^+^ CTL within islets respond to IL-21

**Figure 4:**
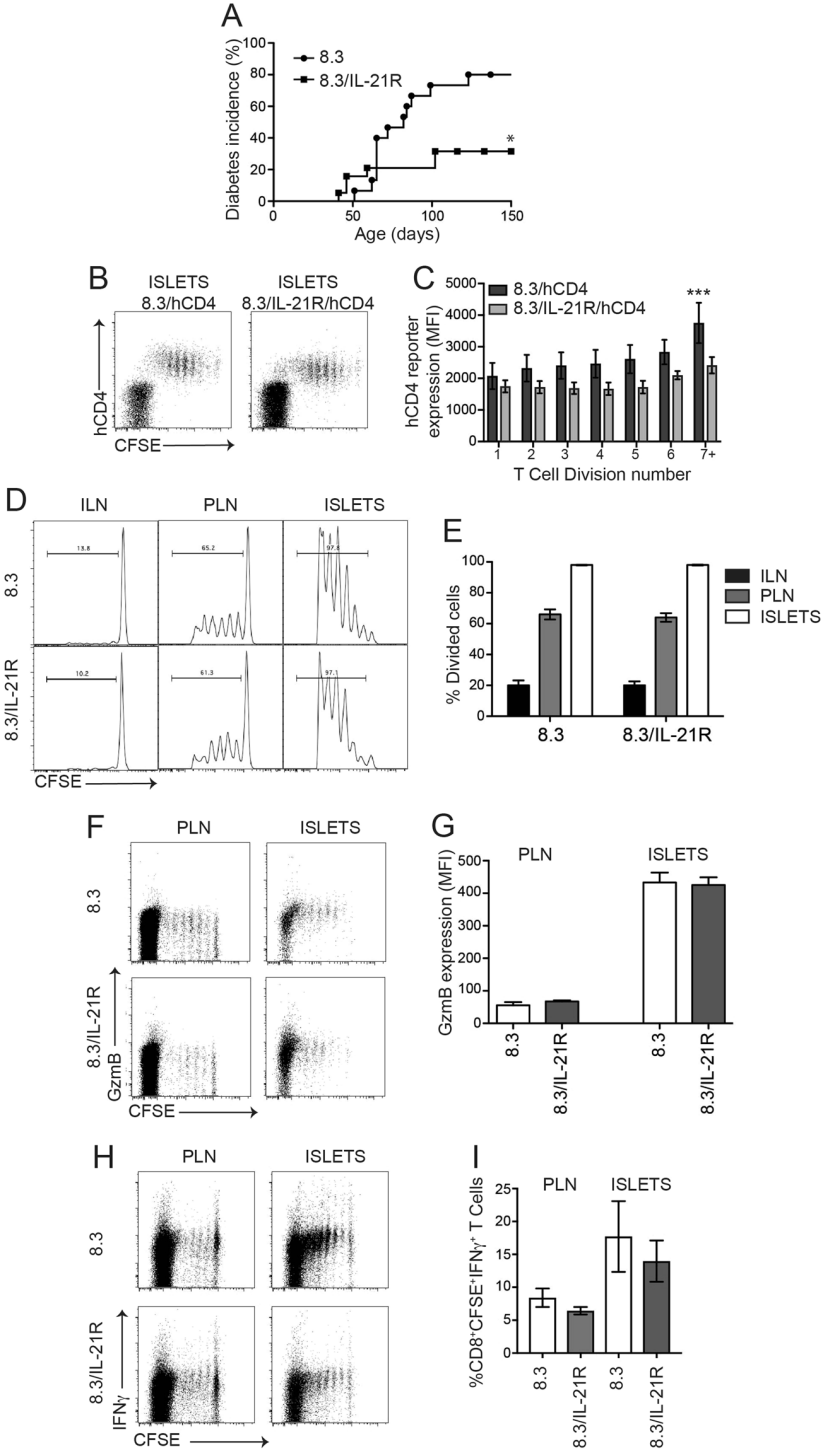
IL-21R is required for maximal SOCS1 upregulation but not CTL differentiation in 8.3 CD8^+^ T cells in NOD islets. (**A**) Diabetes incidence in 8.3/IL-21R and 8.3 controls (n = 15–19 mice per group, p < 0.05 Log-rank (Mantel-Cox) test). (**B**) Representative plots from islets isolated 5 days after 8.3/IL-21R/hCD4 splenocyte transfer showing expression of hCD4 and CFSE dilution. (**C**) Expression of hCD4 (MFI) on CD8^+^CFSE^+^ T cells for each cell division calculated using CFSE dilution profiles. Data show mean ± SEM for n = 3–8 mice from 3 independent experiments. Difference between genotypes ***p < 0.001 two-way ANOVA. Difference between MFI in division 1 and MFI in division 7 for wild-type p = 0.02, one-way ANOVA with Sidak’s post-test for multiple comparisons. (**D**) Representative histograms of inguinal lymph node (ILN), pancreatic lymph nodes (PLN) and islets isolated 5 days after 8.3/IL-21R splenocyte transfer showing CFSE dilution. (**E**) Mean ± SEM frequency of CD8^+^ T cell division in inguinal lymph nodes, pancreatic lymph nodes and islets of n = 12 mice from 3 independent experiments. Genotypes not statistically different. (**F**) Representative plots of pancreatic lymph nodes and islets isolated 5 days after 8.3/IL-21R splenocyte transfer showing expression of granzyme B and CFSE dilution. (**G**) Expression of granzyme B (MFI) on CD8^+^ T cells in pancreatic lymph nodes and islets. Data show mean ± SEM of n = 8 mice from 4 independent experiments. Genotypes not statistically different. (**H**) Representative plots of pancreatic lymph nodes and islets isolated 5 days after 8.3/IL-21R splenocyte transfer, restimulated with IGRP for 6 hours *in vitro* showing expression of IFNγ and CFSE dilution. (**I**) Frequency of CD8^+^CFSE^+^IFNγ^+^ cells (%) in pancreatic lymph nodes and islets. Data show mean ± SEM of n = 5–10 mice from 3 independent experiments. Genotypes not statistically different.

IL-21 and its receptor IL-21R are critical for the development of autoimmune diabetes in NOD mice and several studies have indicated that this is due in part to its effects on CD8^+^ T cells^19,23,35,47^. IL-21 also activates SOCS1 expression in CD8^+^ T cells^48^. To test if IL-21R is required for islet CD8^+^ T cell function we crossed NOD8.3 mice to NOD/IL-21R knockout mice to generate NOD.8.3 mice deficient for IL-21R (NOD8.3/IL-21R). Diabetes incidence in female NOD8.3/IL21R mice was significantly lower than wildtype NOD8.3 mice (Fig. 4A), in accordance with the previously observed protection in IL-21 or IL-21R deficient NOD mice^16–19^. We then crossed NOD8.3/hCD4 mice to NOD/IL-21R mice to generate NOD8.3/IL-21R/hCD4 to determine whether IL-21R signalling is required for CTL differentiation *in vivo*. CFSE labelled T cells from NOD8.3/hCD4 or NOD8.3/IL-21R/hCD4 mice were adoptively transferred into 12–15 week old NOD mice and analysed after 5 days (Fig. 4B). SOCS1/hCD4 reporter expression was reduced in pancreatic islet-infiltrating CTL that lacked IL-21R, suggesting that islet CD8^+^ CTLs upregulate SOCS1 in response to IL-21 (Fig. 4C). However, the proliferation of transferred NOD8.3/IL-21R T cells was not affected by loss of IL-21R (Fig. 4D,+E). In addition, granzyme B expression and IFNγ production were not impaired by loss of IL-21R signalling in transferred NOD8.3/IL-21R T cells in pancreatic lymph nodes or islets (Fig. 4F–I). Together these data indicate that islet CTL respond to IL-21, resulting in upregulation of the SOCS1/hCD4 reporter after transfer, however IL-21R expression on islet CD8^+^ T cells is not required for CTL proliferation or differentiation, as determined by upregulation of granzyme B and IFNγ expression.

## Discussion

Together our experiments provide further insight into cytokine signalling in islet CD8^+^ T cells in the NOD model of T1D. SOCS1 reporter expression increases in beta cells as the islets become infiltrated during the insulitis phase, consistent with previous findings showing IFNγ action on beta cells of infiltrated islets^36^. Our data also indicate that CTLs are exposed to IL-21 in islets and that this contributes in a non redundant way to SOCS1 expression in islet CD8^+^ CTLs. However these IL-21 signals are dispensible for islet CTL maturation. In contrast IFNAR1 was completely dispensible for SOCS1 upregulation and islet CTL maturation events.

Recent data provides a compelling case for IL-21 as a critical immune regulator of T1D pathogenesis, since IL-21 and IL-21R deficient NOD mice are almost completely protected from diabetes, depending on the animal house^16–19^. Additionally IL-21 blocking antibodies are able to reverse recent onset diabetes in NOD mice^49^. While it is clear that loss of IL-21R can affect a number of immune lineages including CD4^+^ and CD8^+^ T cells, B cells and dendritic cells, it is reasonable to speculate that CD8^+^ T cells may be an important locus of IL-21 effects for the development of T1D. IL-21 promotes CD8^+^ CTL development *in vitro*^23,50^ and IL-21R deficient CD8^+^ T cells have impaired diabetogenic function in NOD mice^35,47^. Given these data we hypothesised that IL-21R signals would be an essential islet intrinsic CTL maturation signal. While 8.3 IL-21R mice are protected from autoimmune diabetes, in keeping with phenotypes of IL-21 and IL-21R deficient NOD mice, surprisingly IL-21R deficient 8.3 CD8^+^ T cells have no obvious defects in proliferation or CTL maturation in the islets of NOD mice. Together our data indicate that the CD8^+^ T cell dysfunction in IL-21R deficient NOD mice is unlikely to result from impaired CD8^+^ T cell priming or CTL maturation.

IL-21 regulates the formation of memory CD8^+^ T cells^51,52^ and is required for their optimal function in recall responses in certain viral models^53–55^ suggesting that the absence of IL-21R on CD8^+^ T cells may impair the development or function of the islet antigen specific CD8^+^ T cell memory pool in NOD mice. Additionally IL-21 is important for preventing CD8^+^ T cell exhaustion in chronic viral infections^24,56–58^ suggesting that it may have similar effects in the context of T1D. Thus the CD8^+^ intrinsic defects that are apparent in IL-21R deficient NOD mice may occur after the initial processes of CD8^+^ T cell priming and CTL maturation.

Type I IFNs have been implicated as one important CTL maturation signal, predominantly in the context of viral infections. IFNα can drive CTL maturation in CD8^+^ T cells, as demonstrated by upregulation of granzyme B, IFNγ, and FasL^15,59^, and IFNAR1 knockout CD8^+^ T cells have impaired granzyme B expression^45^. Additionally type I IFNs can promote migration of CD8^+^ T cells into islets^12^. While NOD mice exhibit a prominent IFN induced gene signature during the time course of disease progression^60^ and anti IFNAR1 antibodies protected NOD mice from autoimmune diabetes^13^, our data indicate that IFNAR1 is not required for the development of autoimmune diabetes in NOD mice^34^. In keeping with those findings we show that IFNAR1 deficient 8.3 mice also have normal diabetes incidence and CD8^+^ T cells have normal levels of granzyme B expression after transfer and isolation from NOD islets. These data indicate that type I IFNs are not required for islet CD8^+^ CTL maturation or diabetes onset in 8.3 NOD mice.

In this study we have explored the mechanisms underpinning the islet specific CTL maturation events that are evident in NOD mice. Our data indicate that CTLs are likely to be under the influence of SOCS1 activating cytokines in NOD islets, however neither of the two candidate cytokine receptors tested, IFNAR1 and IL-21R, are necessary for CTL maturation to occur in islets. This may imply that this process is independent of SOCS1 activating effects, that other cytokines are more critical regulators of the maturation processes or that type I IFNs and IL-21 are functionally redundant in this context.

## Supplementary information

Supplementary Figure 1
