# Supplementary material for: IL-21 regulates SOCS1 expression in autoreactive CD8+ T cells but is not required for acquisition of CTL activity in the islets of non-obese diabetic mice
Source: Sci Rep. 2019 Oct 25;9:15302. doi: 10.1038/s41598-019-51636-5 (PMC6814838; doi:10.1038/s41598-019-51636-5)
Supplement: Supplementary file 1 — Supplementary Figure 1 [file 41598_2019_51636_MOESM1_ESM.pdf]

IL-21 regulates SOCS1 expression in autoreactive CD8^{+} T cells but is not required for acquisition of CTL activity in the islets of non-obese diabetic mice

Andrew P. R. Sutherland[1,2*], Kate L. Graham[1,2*], Michelle Papadimitriou[1], Gaurang Jhala[1,2], Prerak Trivedi[1,2], Tara Catterall[1], Stacey Fynch[1], Thomas W. H. Kay[1,2], Helen E. Thomas[1,2]

[1]St Vincent's Institute of Medical Research, Melbourne, Australia

[2]The University of Melbourne, Department of Medicine, St. Vincent's Hospital, Fitzroy, Victoria, Australia

*Authors contributed equally

Address correspondence to:
A/Prof Helen Thomas
St. Vincent's Institute of Medical Research, 9 Princes Street, Fitzroy, VIC 3065, Australia.
E-mail address: hthomas@svi.edu.au
Or
Dr. Andrew P. R. Sutherland
St. Vincent's Institute of Medical Research, 9 Princes Street, Fitzroy, VIC 3065, Australia.
E-mail address: asutherland@svi.edu.au

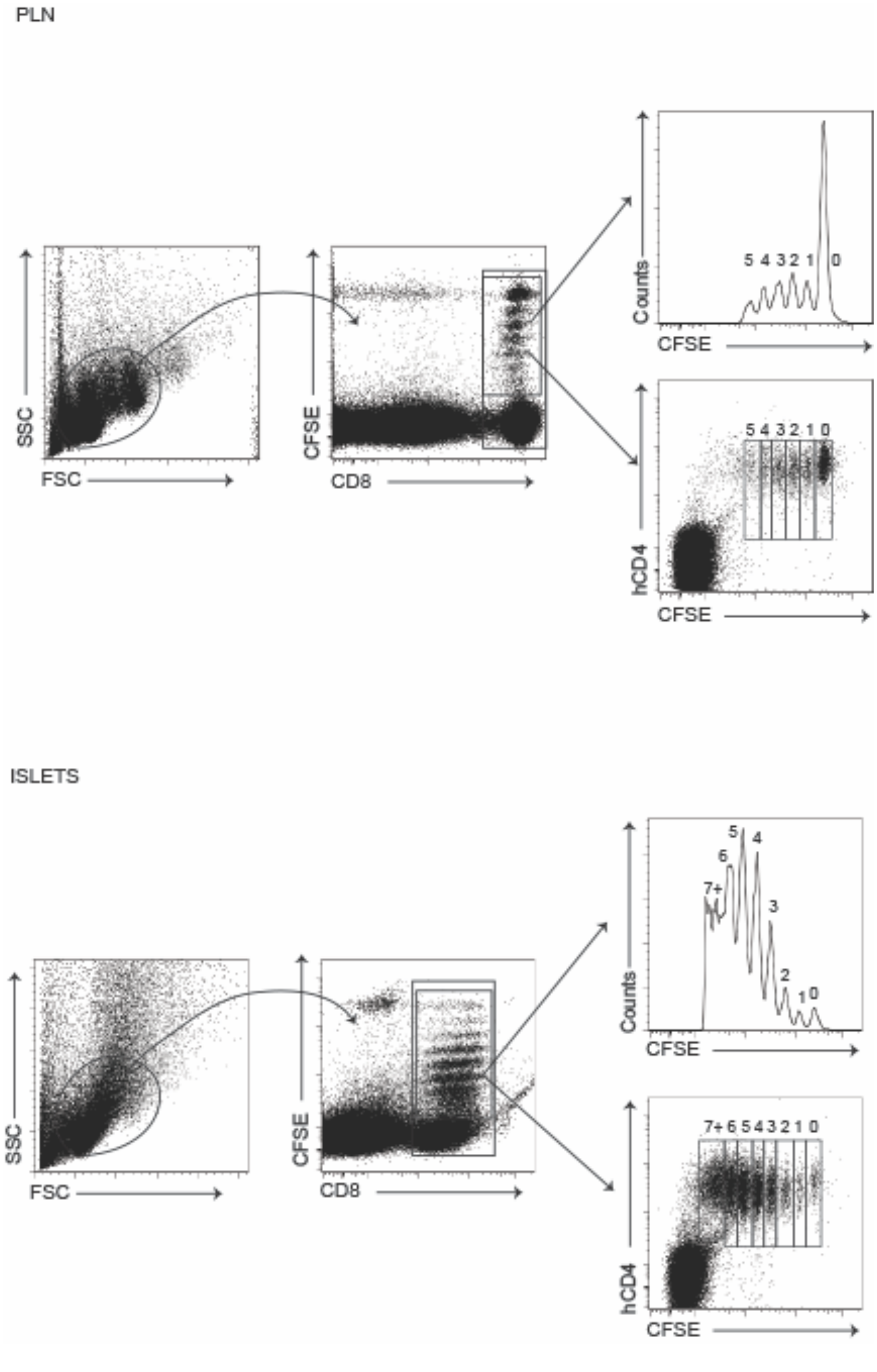

<u>Supplementary Figure 1</u>: ***Quantification of CFSE dilution and hCD4 reporter expression in transferred 8.3 T cells in NOD pancreatic lymph nodes and islets.***

Pancreatic lymph nodes and islets were isolated 5 days after 8.3 splenocyte transfer and

anti CD8 antibody staining and CFSE labelling used to identify the transferred 8.3 T cells.
